# Supplementary material for: Development of Digital Droplet PCR Targeting the Influenza H3N2 Oseltamivir-Resistant E119V Mutation and Its Performance through the Use of Reverse Genetics Mutants
Source: Curr Issues Mol Biol. 2023 Mar 17;45(3):2521–32. doi: 10.3390/cimb45030165 (PMC10047791; doi:10.3390/cimb45030165)
Supplement: Supplementary file 1 [file cimb-45-00165-s001.zip › cimb-2243738-supplementary.pdf]

Supplementary data

Table S1: Primers used for Sanger sequencing to verify the sequences of the mutant A(H1N1)pmd09 and A(H3N2) NA-plasmids

**pRF-CMV Fw**	GATAGCGGTTTGACTCACG
**H3N2sNA-2f**	GCAAAAGCAGGAGTAAAGATGAA
**H3N2sNA-418f**	CCTTGGACAGGGAACAAC
**N2-550f**	ATGGTCCAGCTCAAGTTGTCA
**N2-645R**	CCATCGTAAATGAAGCTAGC
**H3N2sNA-1436r**	CGAAAGCTTATATAGGCATGAGA
**pRF-polyA-Rv**	CTCTAGCATTTAGGTGACC

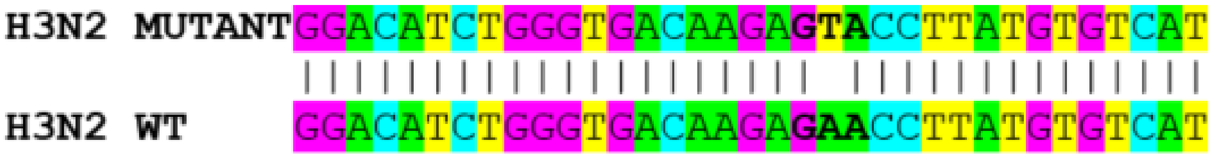

Figure S1: Results of sequence of mutant and wild-type H3N2 around the mutation E119V (GAA > GTA)

Table S2: Mean results for the RT-ddPCR and the phenotypic test

Mixes	Expected		RT-ddPCR		Phenotypic (IC_{50})
	WT	Mutant	WT	Mutant	
**Mix1**	0	100	0.21 ± 0.01%	99.79 ± 0.01%	1.0 ± 0.0
**Mix2**	10	90	-	-	0.9 ± 0.4
**Mix3**	20	80	-	-	1.1 ± 0.6
**Mix4**	30	70	-	-	1.2 ± 1.2
**Mix5**	40	60	-	-	1.1 ± 0.8
**Mix6**	50	50	41.90 ± 0.31%	58.10 ± 0.31%	0.7 ± 0.5
**Mix7**	80	20	60.02 ± 2.16%	39.98 ± 2.16%	0.9 ± 0.8
**Mix8**	90	10	75.69 ± 2.60%	24.33 ± 2.60%	4.8 ± 3.5
**Mix9**	95	5	85.86 ± 1.13%	14.14 ± 1.13%	11.6 ± 12.2
**Mix10**	99	1	97.03 ± 0.48%	2.97 ± 0.48%	64.1 ± 35.1
**Mix11**	99.5	0.5	98.40 ± 0.13%	1.60 ± 0.13%	117.7 ± 6.7
**Mix12**	99.9	0.1	99.60 ± 0.04%	0.40 ± 0.04%	158.8 ± 14.0
**Mix13**	100	0	99.88 ± 0.08%	0.12 ± 0.08%	177.6 ± 24.2

Table S3: Raw ddPCR data and phenotypic test data

Mix	Expected MT%	RT-ddPCR							Phenotypic test
		E119 (copies/µL)	V119 (copies/µL)	Accepted droplets	Negative droplets	E119+ droplets	V119+ droplets	E119+/V119+ droplets	Fold change (IC_{50})
**1**	100	2.5	2980	9805	774	3	9010	18	167.5
**1**	100	1.8	2640	8693	916	3	7764	10	178.9
**1**	100	2.5	4650	7006	135	0	6856	15	186.5
**2**	90	-	-	-	-	-	-	-	165.5
**2**	90	-	-	-	-	-	-	-	168.2
**2**	90	-	-	-	-	-	-	-	142.8
**3**	80	-	-	-	-	-	-	-	123.1
**3**	80	-	-	-	-	-	-	-	117.5
**3**	80	-	-	-	-	-	-	-	112.4
**4**	70	-	-	-	-	-	-	-	97.0
**4**	70	-	-	-	-	-	-	-	68.2
**4**	70	-	-	-	-	-	-	-	27.2
**5**	60	-	-	-	-	-	-	-	25.0
**5**	60	-	-	-	-	-	-	-	8.8
**5**	60	-	-	-	-	-	-	-	1.0
**6**	50	1373	3890	8727	106	213	2611	5797	8.7
**6**	50	507.2	779.4	9653	2431	2318	3653	1251	4.0
**6**	50	247	724	9471	4203	916	3476	876	1.6
**7**	20	614	329	5973	2676	1839	868	590	0.0
**7**	20	232	156	8486	6120	1313	849	204	1.5
**7**	20	1177	699	6186	11239	2176	1036	1735	1.1
**8**	10	662	150	7756	3902	2925	518	411	0.2
**8**	10	555	151	9465	5222	3105	683	455	1.2
**8**	10	1031	286	9579	3140	4373	847	1219	0.6

**9**	5	874	99	5875	2558	2842	236	239	0.3
**9**	5	798	111	8963	4170	3987	377	429	1.2
**9**	5	1044	115	8769	3256	4699	355	459	1.9
**10**	1	1074	18.3	5310	2089	3139	42	40	2.4
**10**	1	1019	24.8	12080	4977	6851	104	148	1.2
**10**	1	1042	21	7859	3184	4536	58	81	0.1
**11**	0.5	1062	11.1	6618	2662	3894	21	41	1.7
**11**	0.5	746	9.9	9698	5102	4515	44	37	1.1
**11**	0.5	1153	11.3	12937	4814	7999	42	82	0.5
**12**	0.1	1169	2.8	9621	3553	6045	8	15	0.5
**12**	0.1	794	2.2	8507	4322	4169	9	7	1.0
**12**	0.1	1016	3.1	7257	3054	4184	7	12	1.2
**13**	0	730	1.2	9022	4842	4171	7	2	1.0
**13**	0	1210	0.59	12047	4304	7737	3	3	1.0
**13**	0	1088	0.5	7612	3018	4591	0	3	1.0

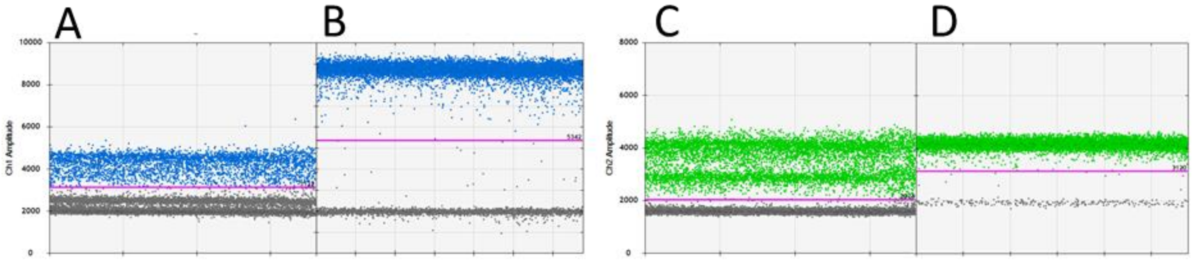

Figure S2: 1D view in QuantaSoft of results on optimization quencher. These figures show the difference in discriminating the viral populations before and after optimization of the quencher and replacing the BHQ-1 (A, C) quencher by a MGB-quencher (B, D). The negative droplets are shown in black, while droplets containing the FAM-labelled (A, B) E119-wild-type are blue and the HEX-labelled (C, D) V119-mutant are green

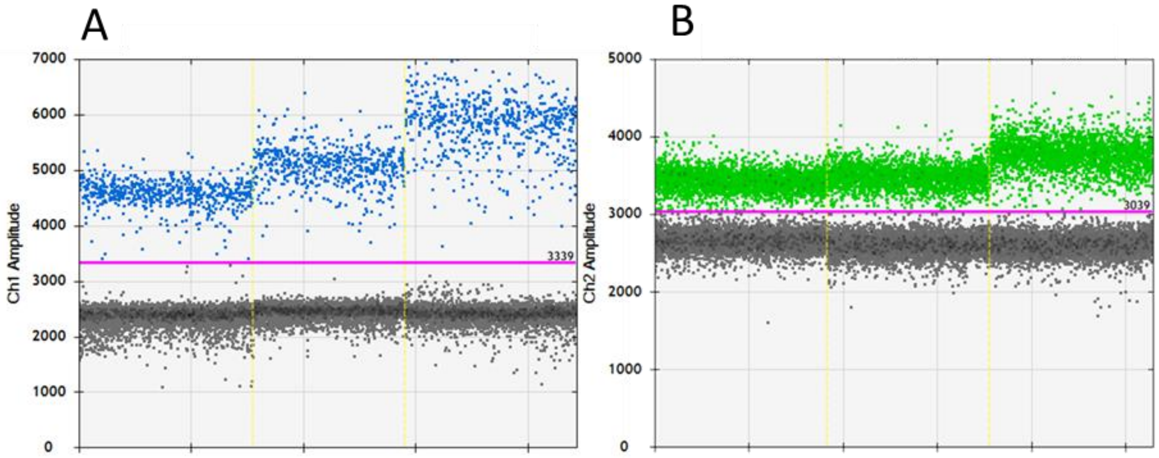

Figure S3: 1D view in QuantaSoft of results on optimization of the annealing temperature using a thermal gradient. These figures show the difference in discriminating the viral populations using a thermal gradient ranging from 58°C to 54.8°C (samples from left to right: 58°C, 56.5°C, 54.8°C). The negative droplets are shown in black, while droplets containing the FAM-labelled (A) E119-wild-type are blue and the HEX-labelled (B) V119-mutant are green.
